# Editorial: Molecular Bridges Between Hematology and Inflammation

**DOI:** 10.3389/fmed.2019.00017

**Published:** 2019-02-08

**Authors:** Ana Kasirer-Friede

**Affiliations:** Department of Medicine, University of California, San Diego, San Diego, CA, United States

**Keywords:** vascular, immune, inflammation, hemostasis, clotting

The topic of Molecular Bridges between Hematology and Inflammation transcends the simplistic classification of blood cells as immune/inflammatory cells (leukocytes) or hemostatic ones (platelets), and additionally factors endothelial contributions into complex hematological and inflammatory processes.

The articles in this compendium describe the close molecular communications that exist amongst vascular and circulatory cells. Platelets play multifaceted roles in injury, inflammation, and restorative remodeling. Despite their anucleate state, or rather because of their lack of genetic plasticity, they provide biological information to instruct close and distant neighbors: various platelet surface receptors, such as αIIbβ3, FcγRIIA, toll-like receptors, DC-SIGN, and CLEC-2 mediate binding not only to plasma ligands for hemostasis, but promiscuously, additionally react with innate and adaptive immune cell receptors, and microbial pathogens. Platelet storage granules contain close to 300 soluble mediators that are released in a regulated fashion upon activation, and include adhesion molecules, bacterial defensins and antiviral agents, growth factors and cytokines. These may disseminate signals for smooth muscle cell proliferation, and promote inflammation by activation of transcriptional modules promoting leukocyte plasticity and upregulation of their reactive receptors and molecules. Engagement of platelet extracellular receptors may additionally induce platelet P-selectin mobilization to the plasma membrane for interaction with endothelial cell or neutrophil PSGL-1, or trigger release of platelet microvesicles, that are borne through the bloodstream and available for cellular endocytosis. Leukocytes, in turn, may impact the hemostatic system by influencing both coagulation and fibrinolysis through expression of tissue factor or plasminogen receptors and, under proinflammatory conditions, may release procoagulant mediators such as cathepsin G and elastase. Furthermore, leukocytes can migrate through thrombi, and phagocytose apoptotic cells and thrombus debris. The four articles below discuss molecular communicators originating from classical hemostatic cells, platelets, or from immune/inflammatory cells, whose functions extend beyond their cellular classifications.

Hottz et al. provide an extensive review of the role of platelets in the immune response to viral infections. Figure 1, illustrates various pathways of viral interaction and internalization involving diverse platelet receptors more often associated with immune cells, such as DC-SIGN, C-type lectin family receptors (CLEC), and toll-like receptors (TLR). The authors focus on three viruses that impact global health: HIV, responsible for AIDS, influenza, and Dengue (DENV) virus, that causes a hemorrhagic disease. Throughout, discussions of platelet activation in the pathophysiology of viral infections highlight elicited changes in platelet ultrastructure, signaling networks, and granule release, including of antivirals RANTES/CCL5 and PAF/CXCL4. Viruses may breach normal barriers to infection, e.g., the alveolar-capillary barrier, and foster platelet infiltration of lungs or other tissue, potentially leading to microvascular thrombosis and secretion of inflammatory mediators. The authors describe recent studies of novel feedback loops between DENV, neutrophils, and platelets involving cell-free histone 2A recognition by TLR2 and TLR4 on platelets, and association of platelets and neutrophils with cell-free histones in areas of alveolar damage in influenza infected mice.

Eisinger et al. develop the premise that tissue injury leads to release of platelets' inflammatory α-granule cargo, to trigger recruitment and activation of inflammatory cells. In particular, sCD40L may upregulate monocyte tissue factor expression and coagulation, or EC expression of E-Selectin, ICAM1, and VCAM1, which together with platelet-released RANTES binding to inflamed endothelium, can increase leukocyte recruitment and transmigration. Furthermore, granule cargo may influence the regenerative capacities of ECs: it is a common theme that granule molecules both promote and inhibit cellular activity, and accordingly, the pro-angiogenic mediators VEGF, MMPs, PDGF, SDF-1 are released, as is anti-angiogenic thrombospondin. The authors provide several examples of how the immune/inflammatory and hemostatic systems may interact, as illustrated by the following. Tissue injury accompanied by neutrophil infiltration, with release of NETS and histones, may strongly stimulate platelets, and when teamed with platelet-released polyphosphates, may enhance platelet procoagulant activity by 20-fold. Similarly, increased thrombogenicity of platelet-derived high mobility-group box 1 protein (HMGB-1) when oxidized by ROS released from recruited monocytes, may act to support inflammation-induced venous thrombosis.

Mosevoll et al. approach thromboinflammation from the premise that inflammatory biomarkers and chemical mediators drive inflammatory venous thromboembolism, primarily through cytokines, adhesion molecules, and matrix metalloproteases (MMP). The authors discuss the increases in levels of cytokines IL-6, IL-8/CXL-8, TNFα in this condition. They introduce the idea of post thrombotic syndrome (PTS), where residual levels of MMPs may affect thrombus resolution. Two tables are presented that list molecules of interest in the development, propagation and aggravation of venous thrombosis: the first lists cytokines that may function as predisposing factors, diagnostic markers and prognostic markers, and the second summarizes relevant leukocyte adhesion molecules. The authors suggest tracking these molecules in thromboinflammation, and that by developing anti-cytokine therapeutics, it may be possible to alleviate the inflammatory response, and ameliorate thrombus resolution in post thrombotic syndrome development.

Tseng et al. present an original research paper that discusses cell-cell interactions in sterile inflammation. Neutrophil-generated reactive oxygen species (ROS) such as NADPH oxidase 2 have been established to regulate the function of αMβ2 in platelet-neutrophil interactions, that on activated ECs, may mediate microvascular occlusion and tissue damage under sterile inflammatory conditions. Interestingly, in the present study, using *in vitro* transmigration assays and an *in vivo* ischemia/reperfusion-induced hepatic injury model, the authors find that while MPO-derived oxidants may negatively regulate αMβ2 and hence neutrophil adhesive and migratory functions, no MPO-driven changes in platelet-neutrophil interactions were observed, suggesting selectivity in ROS-induced heterocellular interactions. The loss of MPO, however, reduced tissue damage compared to WT mice, and suggested that reduced inflammation would nevertheless have an indirect effect on thrombo-inflammatory reactions. Based on these results, the authors propose that MPO might be an attractive therapeutic target for the treatment of numerous inflammatory diseases.

These articles deliver a closer understanding of how cell-of-origin molecular species may hijack, restructure, or otherwise modify other blood cells through effects on inflammation, angiogenesis and coagulation, and will expand the potential for development of non-canonical diagnostics or interventions.

## Author Contributions

The author confirms being the sole contributor of this work and has approved it for publication.

### Conflict of Interest Statement

The author declares that the research was conducted in the absence of any commercial or financial relationships that could be construed as a potential conflict of interest.

